# Engineering the Catalytic Properties of Two-Domain Laccase from *Streptomyces griseoflavus* Ac-993

**DOI:** 10.3390/ijms23010065

**Published:** 2021-12-22

**Authors:** Ilya Kolyadenko, Anastasia Scherbakova, Kirill Kovalev, Azat Gabdulkhakov, Svetlana Tishchenko

**Affiliations:** 1 Institute of Protein Research RAS, 142290 Pushchino, Russia; nas.scherbakova99@bk.ru (A.S.); azat@vega.protres.ru (A.G.); sveta@vega.protres.ru (S.T.); 2 European Molecular Biology Laboratory, 22607 Hamburg, Germany; kirkovalev94@gmail.com; 3 Research Center for Molecular Mechanisms of Aging and Age-Related Diseases, Moscow Institute of Physics and Technology, 141701 Dolgoprudny, Russia

**Keywords:** two-domain laccases, crystal structures, site-directed mutagenesis, proton wire, substrate-binding site, catalytic activity

## Abstract

Laccases catalyze the oxidation of substrates with the concomitant reduction of oxygen to water. Recently, we found that polar residues located in tunnels leading to Cu2 and Cu3 ions control oxygen entrance (His 165) and proton transport (Arg 240) of two-domain laccase (2D) from *Streptomyces griseoflavus* (SgfSL). In this work, we have focused on optimizing the substrate-binding pocket (SBP) of SgfSL while simultaneously adjusting the oxygen reduction process. SgfSL variants with three single (Met199Ala, Met199Gly, and Tyr230Ala) and three double amino acid residues substitutions (Met199Gly/His165Ala, His165Ala/Arg240His, Met199Gly/Arg240His) were constructed, purified, and investigated. Combination of substitutions in the SBP and in the tunnel leading to Cu2 ion (Met199Gly/Arg240His) increased SgfSL catalytic activity towards ABTS by 5-fold, and towards 2.6-DMP by 16-fold. The high activity of the Met199Gly/Arg240His variant can be explained by the combined effect of the SBP geometry optimization (Met199Gly) and increased proton flux via the tunnel leading to Cu2 ion (Arg240His). Moreover, the variant with Met199Gly and His165Ala mutations did not significantly increase SgfSL’s activity, but led to a drastic shift in the optimal pH of 2.6-DMP oxidation. These results indicate that His 165 not only regulates oxygen access, but it also participates in proton transport in 2D laccases.

## 1. Introduction

Laccases (EC 1.10.3.2) are multi-copper oxidases that catalyze the oxidation of various organic and inorganic molecules (e.g., mono- and di-phenols, polyphenols, diamines, aminophenols, aromatic or aliphatic amines) with the reduction of molecular oxygen to water [1,2]. Laccases are important industrial catalysts, being widely used in paper and pulp, cosmetics and pharmaceuticals, textile, food and feed sectors, biocatalysis, polymer and synthetic chemistry, and even in materials science [3].

Since its discovery in 1883, laccases have been found in higher plants, some insects, fungi, lichens, bivalves, crustaceans, bacteria, and archaea [4]. Typically, laccases are three-domain (3D) proteins, but among bacterial laccases, two-domain (2D) enzymes can also be found. Bacterial 2D laccases show interesting biophysical properties, including activity and stability over a broad range of pH and high temperature, and resistance to chemical inhibitors. Nevertheless, compared to 3D laccases, their activity is lower, especially towards massive substrates with high redox potential. Still, as the industrial application potential of 3D laccases is limited by their narrow temperature and pH stability, and increased sensitivity to organic solvents and inhibitors [5,6], focus has been put into studying and engineering of 2D laccases. 

Both 2D and 3D laccases harbor four copper ions in active centers (Cu1, Cu2, and Cu3α-Cu3β). The structure, geometry, and residues of 1st coordination sphere of such ions are the same in both laccases, but the amino acids of the 2nd coordination sphere differ significantly. A Cu1 ion is coordinated by two histidine residues, and a cysteine residue with a fourth variable axial ligand. Usually, methionine is the axial ligand of bacterial laccases, while phenylalanine or leucine are found in their fungal counterparts. Substrate oxidation occurs near the Cu1 ion (named T1 center), and electrons are then transferred via the Cys-His pathway to the Cu2 and Cu3α-Cu3β (TNC), where the reduction of oxygen to water takes place [7]. Thus, the T1 center of laccases is the primary electron acceptor and the substrate-binding site located near the Cu1 ion. The TNC center of laccases is located buried inside the protein, but it connects to the surface via the T2 and T3 tunnels. 

A few structures of laccase-substrate complexes have been solved. However, the resolution of the structures is generally low, and ligand atoms have high values of temperature factors [8,9,10]. One of the most reliable structural data available is that of 3D laccase from *Melanocarpus albomyces* in complex with 2.6-DMP (PDB id 3FU9). In this structure, only one of the two histidines belonging to the 1st coordination sphere of Cu1 ion forms a hydrogen bond with an oxygen atom of the phenol compound. Nevertheless, other residues of the SBP stabilize the complex by hydrophobic interactions. Besides, negatively charged aspartic acid residue nearby the Cu1 is also supposed to be a proton acceptor during phenols oxidation [9]. Altogether, this indicates that one of Cu1 coordinating histidine residues is the principal electron acceptor in the oxidation reaction, and that residues of the 2nd coordination sphere are responsible for the orientation of the substrate in the SBP, thus influencing the laccases substrate affinity and catalytic rate.

The reactivity of laccases depends on the redox potential (RP) of a Cu1 ion; for bacterial laccases, it usually does not exceed 500 mV, whereas it can go up to 800 mV for more active fungal laccases. To date, there is no consensus on how the structure and immediate environment of a Cu1 ion affect its redox potential and catalytic activity. The substitutions of axial Met of the 3D bacterial laccase CotA to Leu and Phe increased the redox potential by approximately 50–100 mV, but the catalytic activity of the mutant variants was lower than that of the native enzyme [11]. The mutation of axial Leu of 3D laccase *M. albomyces* to Met resulted in a decrease of the enzyme’s RP by 140 mV, which impacted its activity toward ABTS and 2.6-DMP [12]. Substitution of the Cu1 axial ligand Met510Leu in CueO (*Escherichia coli*) in combination with the mutation of Cu1 2nd coordination sphere residue Asp439Ala, led to increasing enzyme activity toward ABTS by more than 100-fold; however, the RP of the enzyme remained practically unchanged [13]. In the 2D laccase Ssl1 from *Streptomyces sviceus*, substitutions of two Met’s residues (axial Met and Met belonging to 2nd coordination sphere of Cu1) decreased the activity toward a phenolic substrate [14]. Alternatively, replacement of the same 2nd coordination sphere Met of 2D laccase SLAC (*S. coelicolor*) by residues with minimal side groups (glycine and alanine), increased the catalytic activity of the laccase to the phenolic substrate, presumably improving substrate access to the T1 center [15]. A set of mutations near the Cu1 ion of Ssl1 increased the RP of the enzyme by 185 mV, but the efficiency of syringaldazine oxidation decreased more than 1000-fold. [16]. Based on these data, we suggest that RP is not the main factor affecting laccases activity.

Since the active center of laccases consists of two parts (T1 and TNC), enzyme activity depends not only on the characteristics and environment of the SBP, but also on the rate of oxygen reduction in the TNC [17]. T3 and T2 tunnels provide molecular oxygen and protons access to TNC, as well as water extrusion. In 3D laccases, two conserved 2nd coordination sphere negatively charged residues located in T3 and T2 tunnels participate in oxygen protonation during the reduction process [17]. One of them is situated close to Cu3β, the other is close to Cu2. Earlier, we have investigated the role of polar residues located in T2 (Arg 240) and T3 (His 165) tunnels in the catalytic activity of 2D laccase from *Streptomyces griseoflavus* (SgfSL). We have shown that His 165 is the “gateway” of the 2D laccase T3 tunnel, and that it probably regulates the access of oxygen to TNC. The His165Ala mutation increased the tunnel size and the enzyme activity 1.5-fold at acidic conditions, using ABTS as substrate [18]. Moreover, replacement of Arg 240 in the T2 tunnel to His increased the turnover number (k_cat_) of SgfSL activity at acidic and alkaline (using 2.6-DMP as substrate) conditions more than 3-fold. Based on these results, we proposed that Arg 240 participates in the proton transfer pathway to TNC through the H-bond network [19]. Thus, optimization of the oxygen reduction process improved the activity of the 2D laccase without changing its SBP.

**Figure 1:**
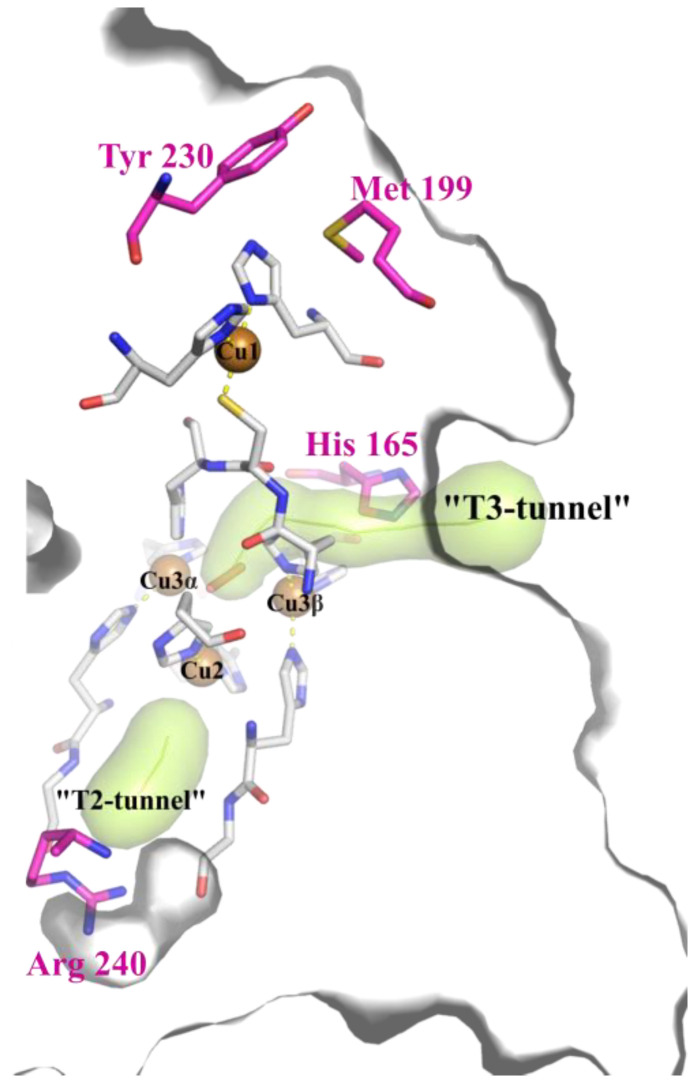
The positions of the substituted amino acids in the SgfSL SBP (Met 199, Tyr 230), near Cu3β (His 165) and Cu2 (Arg 240) copper ions (PDB id 6S0O). Copper ions are shown with brown spheres. Coordinating copper ions amino acids are shown as gray sticks. Side group of Met 199, His 165, and Arg 240 are shown in magenta. T2 and T3 tunnels are shown with green surfaces. The contour of the protein surface is shown in gray. Peroxy intermediate (PER) is depicted with a pale pink stick between the copper ions.

In this work, to create a high-active enzyme, we continued to investigate the role of the T2 and T3 tunnels of 2D laccases, and additionally focused on engineering simultaneously its SBP and TNC, using SgfSL as a model. Three mutants of SgfSL with substitutions of residues belonging to the 2nd coordination sphere of Cu1 ion (Met199Ala, Tyr230Ala, and Met199Gly, Figure 1) were obtained and investigated in detail. We show that substitutions in the SBP increased the activity of SgfSL toward ABTS and 2.6-DMP by 1.5–5-fold. The most active mutant (Met199Gly) was then chosen to create the SgfSL variant with optimized SBP and TNC. The catalytic activity of double-mutant form Met199Gly/Arg240His towards the substrates ABTS and 2.6-DMP was found to increase by 5- and 16-fold, respectively. Both double substitutions His165Ala/Met199Gly and His165Ala/Arg240His slightly improved catalytic properties of SgfSL but kept high resistance to inhibitor (as for His165Ala variant). We assume the side chain of His 165 participates in the protonation of an oxygen-derived product of catalysis, but the main flux of protons enters the TNC via the T2 tunnel.

## 2. Results

### 2.1. Structural Analysis of the Wild Type and Mutant Enzymes

Diffraction data from the crystals of mutant forms, excluding double variant His165Ala/Met199Gly, were collected at 100 K. Diffraction data from the crystal of His165Ala/Met199Gly variant were collected at room temperature. There was no significant difference in the overall protein folds and conformation of amino acid side chains at room temperature and under cryo-conditions. The r.m.s.d. parameters between wild type structure (PDB 6S0O) and Met199Ala, Met199Gly, Tyr230Ala, His165Ala/Arg240His, His165Ala/Met199Gly, and Met199Gly/Arg240His structures never exceeded 0.40 Å. Due to the high resolution in the structure of the double mutant His165Ala/Arg240His, it was possible to identify almost two thousand water molecules. In contrast, in the structures of other mutant forms, the number of water molecules did not exceed one thousand. Increased amounts of water molecules allowed us to more accurately analyze the effect of amino acids in the T2 and T3 tunnels on the SgfSL functioning. The most important parameters of data collection and structures refinements are summarized in Table 1. 

### 2.2. Kinetic Analysis of the Wild Type and Mutant Enzymes

**Figure 2:**
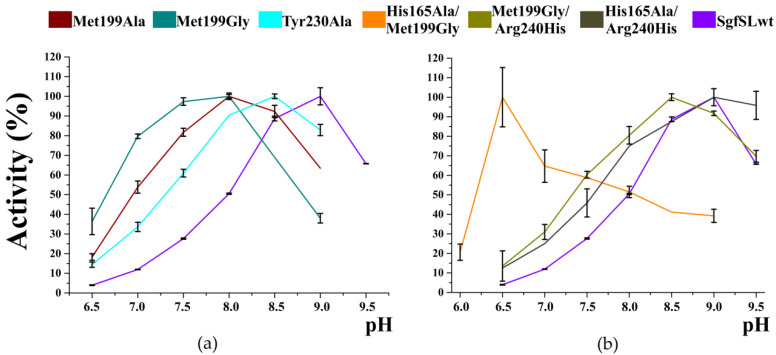
Optimal pH values for 2.6-DMP oxidation by mutant forms and SgfSLwt: (**a**) single mutants, (**b**) double mutants. Error bars indicate the standard deviation for three replicates.

The optimal pH values (pH_opt_) for ABTS and K_4_[Fe(CN)_6_] oxidation were approximately similar between the mutant forms and SgfSLwt (Table 2). In contrast, a significant difference in pH_opt_ of 2.6-DMP oxidation was detected (Table 2 and Figure 2).

Met199Gly and Met199Ala mutations led to a shift of pH_opt_ for 2.6-DMP oxidation by 1.0 point, while Tyr230Ala mutation shifted the pH_opt_ by 0.5 points (Figure 2 and Table 2). The pH_opt_ of 2.6-DMP oxidation of double mutant forms His165Ala/Arg240His and Met199Gly/Arg240His is approximately the same of SgfSLwt. Surprisingly, the optimal pH of His165Ala/Met199Gly variant was pH 6.5, while single-mutant forms Met199Gly and His165Ala exhibited maximum activity at pH 8.0 and 9.0, respectively (Table 2).

Optimization of the SgfSL SBP by the substitutions Met199Gly, Met199Ala, or Tyr230Ala, and combination of these mutations with amino acid replacements close to TNC copper ions (His165Ala and Arg240His), led to a significant increase of ABTS and 2.6-DMP oxidation efficiency (Table 2).

All substitutions led to an increase of SgfSL activity towards ABTS, and almost all mutations increased the enzyme activity towards 2.6-DMP (Table 2). In particular, the Met199Ala mutation nearly doubled the SgfSL activity toward ABTS and 2.6-DMP, and substitution of Met 199 to Gly increased activity of the enzyme toward both organic substrates approximately by 5-fold. The activity of the Tyr230Ala variant toward ABTS and 2.6-DMP was 4 and 2-fold higher than SgfSLwt, respectively. The catalytic efficiencies of double-mutant variants His165Ala/Met199Gly and His165Ala/Arg240His toward ABTS and 2.6-DMP are nearly identical to that observed with single-mutant form His165Ala [15]. Double-mutant Met199Gly/Arg240His exhibited the same catalytic efficiency of ABTS oxidation as Met199Gly, but activity toward 2.6-DMP increased more than 16-fold, compared to SgfSLwt.

In contrast, oxidation of the inorganic substrate K_4_[Fe(CN)_6_] by SgfSL variants showed some what different results. While the catalytic rates observed for all mutants increased, with the exception of His165Ala/Arg240His and His165Ala/Met199Gly, the affinity to the substrate was clearly impaired (Table 2). As a result, the catalytic efficiency of SgfSL variants significantly dropped (Table 2).

### 2.3. Inhibition Assays Using Sodium Azide

The inhibition assays were carried out using ABTS as substrate. The residual activity of SgfSLwt after sodium azide treatment was about 23%. There were no significant differences in sensitivity to sodium azide between SgfSLwt and mutants Met199Ala, Met199Gly, Tyr230Ala, and Met199Gly/Arg240His. However, after sodium azide treatment, the residual activity of His165Ala/Met199Gly and His165Ala/Arg240His variants was higher than that of SgfSLwt (Table 3).

### 2.4. Optimal Temperature and Thermal Stability of SgfSL Variants

**Figure 3:**
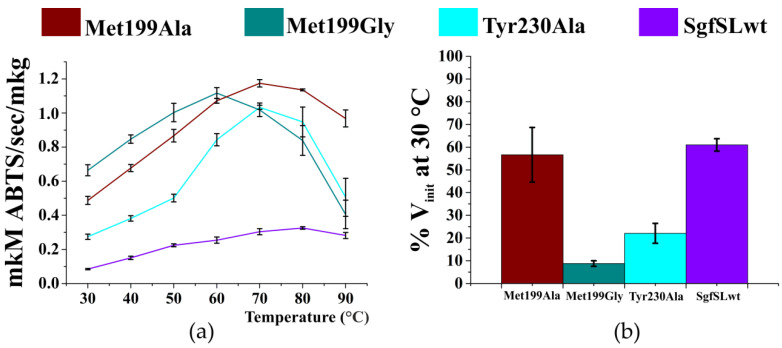
Optimal temperature of ABTS oxidation (**a**) and residual activity after incubation at 80 °C for 30 min (**b**) of the SgfSLwt and mutants Met199Ala, Met199Gly, Tyr230Ala. Error bars indicate the standard deviation for three replicates.

Similarly to the analysis performed with the chemical inhibitor sodium azide, substrate ABTS was used to determine the optimal temperature (T_opt_) and thermal stability of SgfSLwt and respective variants (Figure 3).

The T_opt_ of ABTS oxidation of SgfSLwt is approximately 80 °C. Substitution of Met 199 and Tyr 230 changed T_opt_ values by 10–20 °C (Figure 3a). The most catalytically active variant Met199Gly (Table 2) had maximum activity at 60 °C, whereas Met199Ala and Tyr230Ala have their T_opt_ at 70 °C. In addition, the residual activity of SgfSL mutant forms after incubation at 80 °C for 30 min markedly decreased compared to the wild-type enzyme, except for variant Met199Ala (Figure 3b). SgfSLwt showed close to 60% of initial activity after incubation, while mutant forms Met199Gly and Tyr230Ala exhibited less than 30% of the initial activity under the same treatment.

### 2.5. Comparative Analysis of the Changes in Intensities of Cu1 Absorption Peaks of SgfSLwt and Met199Gly after Heating at 80 °C

**Figure 4:**
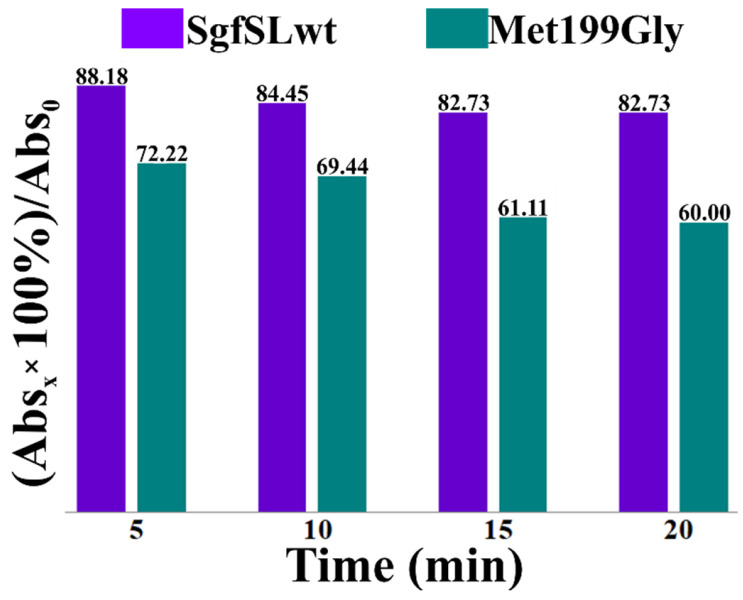
Changes in absorption intensity at 600 nm of SgfSLwt and Met199Gly after heating at 80 °C. The *x*-axis shows the warm-up time. The *y*-axis shows the intensity of the Cu1 ion peak after heating expressed in percentage. Abs_0_—absorbance at 600 nm before the heating; Abs_x_—absorbance at 600 nm after the heat treatment for a specified time.

The absorption peak at λ = 600 nm indicates the presence of Cu1 ion in the T1 center of 2D laccases. To analyze the effect of high temperature on the Cu1 ion absorption peaks of SgfSLwt and Met199Gly, we analyzed the changes in intensity of the peaks during incubation at 80 °C (Figure 4). The initial intensities of the peaks at λ = 600 nm of SgfSLwt and Met199Gly were approximately the same, but after the heat treatment, SgfSLwt retained 83% of initial absorbance, whereas Met199Gly retained only 60%. This indicates that intensity of the Cu1 ion peak of the Met199Gly variant decreased more sharply than SgfSLwt. No protein aggregation was observed during the experiment, as determined by dynamic light scattering analysis (Figure S1).

### 2.6. Decolorization of Industrial Dyes by SgfSLwt and Mutant Forms with Replacements in SBP

**Figure 5:**
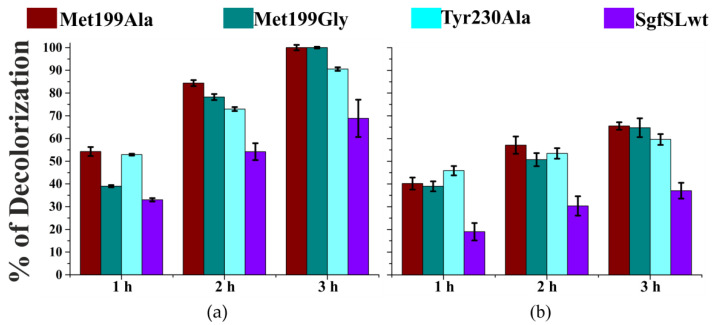
Decolorization of synthetic dyes by SgfSLwt and respective variants over time, using ABTS as mediator: (**a**) indigo carmine, (**b**) malachite green. Error bars indicate the standard deviation for three replicates.

The ability of SgfSLwt and variants Met199Ala, Met199Gly, and Tyr230Ala to decolorize solutions containing two industrially-relevant dyes (indigo carmine and malachite green) was investigated. The assays were performed at a pH optimal for dye decolorization, and with the presence of ABTS as redox mediator. Remarkably, the decolorization rate shown by the mutant-forms compared to the wild-type laccase was increased (Figure 5).

## 3. Discussion

### 3.1. Mutations in the Substrate-Binding Pocket of the 2D Laccase SgfSL Affect Its Activity toward Different Substrates

It has been demonstrated that the reduction potential of Cu1 ion and the difference in reduction potentials of the substrate and the Cu1 are not the only factors determining the activity of 2D laccases [16]. Geometry of the SBP, flexibility of amino acid side chains forming this site, and presence of polar residues may also influence the substrate binding capacity, and thus, the catalytic activity of an enzyme.

**Figure 6:**
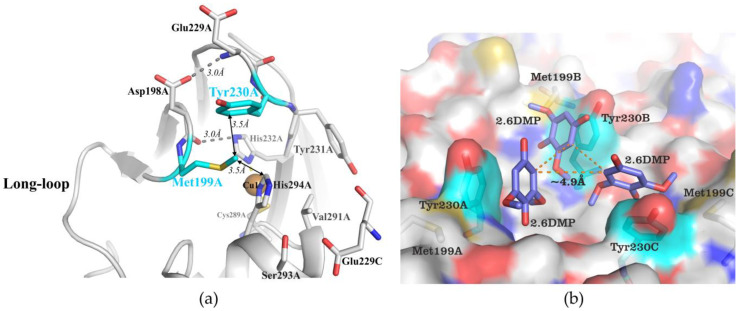
The substrate-binding pocket (SBP) of SgfSL. (**a**) Coordination of the Cu1 ion and its amino acid environment (SBP). Amino acid residues are shown as colored sticks, the copper ion represented as a brown sphere, hydrogen bonds illustrated by dashed lines, and the distance between amino acid residues is shown by lines with arrows. (**b**) Model of 2.6-DMP binding in the SBP of SgfSL. The protein structure is shown by the surface, the substrate and amino acids represented by colored sticks. Dashed lines show the distances between substrate molecules.

The overall structure of all 2D laccases, the amino acid environment, and the structure of their active centers, including substrate-binding pockets, are quite similar. For example, the substrate-binding pocket of SgfSL is a small cavity formed by residues Met 199, Glu 229, Tyr 230, Tyr 231, Val 291, and Ser 293 (numeration by PDB id 6S0O, Figure 6a).

Val 291 and Tyr 231 are located at the bottom of the SBP cavity, whereas other amino acids form the “walls” of this pocket. 2D laccases are homotrimeric proteins, with Cu1 ions of monomers lying in one plane at an equal distance from each other and forming the so-called equilateral triangle. Based on the crystal structure of *M. albomyces* 3D laccase with the substrate 2.6-DMP (PDB id 3FU9), we have done simple molecular dynamics modeling of SgfSL with 2.6-DMP as substrate (Figure 6b). In our opinion, a very close arrangement of T1 centers in the 2D laccase trimer can limit the simultaneous access of bulky substrates (larger than 2.6-DMP) to the binding site.

Based on our structural analysis, we propose that the side chains of Met 199 and Tyr 230 can hinder the access of organic substrates to Cu1 ion (Figure 6a). Supporting this suggestion, we observed that substitution of Met 199 or Tyr 230 to residues with short side chains (Gly, Ala) led to an improvement in rate of catalysis (k_cat_) (Table 2), which represents an increase in the availability of the Cu1 for organic substrates (ABTS and 2.6-DMP). In fact, Met199Gly mutation increased the k_cat_ toward ABTS by 3-fold and by one order of magnitude toward 2.6-DMP, indicating that the SBP geometry change due to Met199Gly mutation appears to be more favorable for oxidation of 2.6-DMP than ABTS (Table 2). For the first time, we have investigated the effect of substitutions in the SBP on the activity of 2D laccases toward inorganic substrates. The efficiency (k_cat_/K_m_) of K_4_[Fe(CN)_6_] oxidation of SgfSL variants is lower than that of the wild-type enzyme. Moreover, affinity to the substrate is also reduced, whereas the oxidation rate is increased (Table 2). It is known that substitution of two Ala’s surrounding the Cu1 ion by hydrophobic Val and Leu improves the activity of fungal laccase (PM1) toward K_4_[Mo(CN)_8_] and decreases affinity to ABTS [20]. In our work, by replacing Met 199 and Tyr 230 the hydrophobicity of SgfSL SBP is likely altered, influencing the enzyme affinity to inorganic substrates. Thus, changing the geometry of the SBP has a different effect on SgfSL activity toward different substrates.

Synthetic dyes are extensively applied in many industries, but their resistance to biodegradation, in addition to their carcinogenic and mutagenic properties, can cause serious environmental problems [3]. The decolorization ability of the mutant variants in the presence of ABTS as mediator is exceeds the activity of SgfSLwt by almost 2-fold (Figure 5). The pH_opt_ for decolorization of the dyes used in this work (indigo carmine and malachite green) lies in the range between 5.0 and 6.5. This is far from the measured mutants’ optimal pH for ABTS oxidation (pH 3.5–4.0). Under the dyes’ decolorization conditions tested here, enzymes exhibit only a small part of their activity. This may partly account for the similar decolorization ability of the mutants variants.

### 3.2. Changing the Geometry of the Substrate-Binding Pocket by Met199Gly and Tyr230Ala Mutations Reduces the Activity of SgfSL after High-Temperature Incubation

Met 199 is located in β-hairpin turning into the loop (203–212 aa), Tyr 230 is in the short loop between two β-strands (Figure 6a). The side chains of both residues contact each other (Met 199CG–Tyr 230CZ, Met 199SD–Tyr 230CE2) and form a side wall of the substrate-binding pocket (Figure 6b). The β-hairpin is stabilized by two hydrogen bonds formed by Asp 198 with the main chain of Glu 229 and with the side chain of His 232 (Figure 6a). Thus, the contacts mentioned above can fix the position of the loop (203–212 aa). 

**Figure 7:**
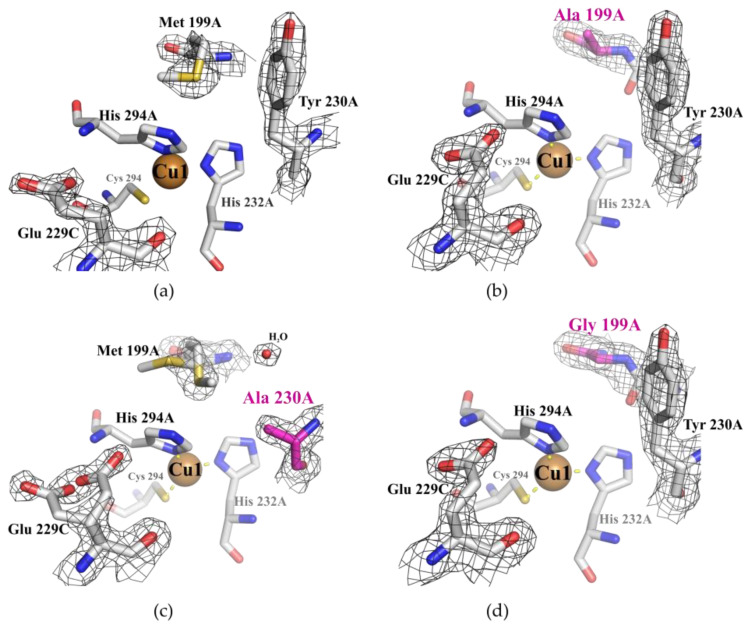
Electron density maps (2Fo-Fc, *δ* = 1) of Cu1 ion environment of SgfSLwt (**a**), Met199Ala (**b**), Tyr230Ala (**c**), and Met199Gly mutants (**d**). Cu1 ions are shown as brown spheres. Gray sticks illustrate Cu1 1st and 2nd coordination sphere amino acids. Positions showing mutations are colored magenta. The water molecule is drawn as a small red sphere. The electron density map is shown as black mesh.

From our point of view, substitutions of Met 199 and Tyr 230 to residues with short side groups (Gly, Ala) can increase the mobility of this region due to loss of contacts strength. Indeed, in the crystal structure of the Tyr230Ala variant, double positions of Met 199 and Glu 229 side chains were found (Figure 7c) unlike the other structures (Figure 7a,b,d). 

We analyzed the residual activity of Met199Ala, Met199Gly, and Tyr230Ala variants after incubation at 80 °C for 30 min (Figure 3). The residual activity of Met199Gly and Tyr230Ala mutants after high-temperature incubation and its optimal temperature (T_opt_) for ABTS oxidation decreased significantly compared to SgfSLwt. The residual activity of the Met199Ala variant after high-temperature incubation was approximately the same as SgfSLwt, but the T_opt_ of ABTS oxidation decreased by 10 °C (Figure 3). We suggest that decreased residual activity of Met199Gly and Tyr230Ala variants at high temperature could be explained by increased mobility of the Cu1 ion environment. As the residual activity of SgfSL due to Met199Ala mutation did not change, the substitution did not affect the mobility of the Cu1 ion environment. At the same time, Met199Ala mutation influenced the T_opt_ of ABTS oxidation, likely due to increased substrate dissociation from the SBP at high temperature.

The position of two (His 294 and His 232) of the three residues belonging to the 1st coordination sphere of Cu1 ion, can strongly influence the position of the loop mentioned above. In the SgfSLwt structure, His 294 is fixed by Met 199, Val 291 and indirectly Tyr 230, whereas His 232 is fixed by H-bond with Asp 198 (Figure 6a). We hypothesize that the proposed loop mobility in mutant variants can disrupt the Asp 198-His 232 and Asp 198 - Glu 229 H-bonds at high temperatures and affect the fixation of Cu1 ion. This hypothesis is based on the changes in intensity of Cu1 ion absorption peaks of SgfSLwt and Met199Gly after high-temperature incubation. We have determined that the intensity of the Cu1 ion peak of the Met199Gly variant in the same incubation conditions decreases more sharply than SgfSLwt (Figure 4). In our opinion, lower peak intensity is associated with the dissociation of Cu1 ions from T1 centers of the enzymes, and not with reduction. These data indicate that mutations Met199Gly and Tyr230Ala may break a well-organized network of non-covalent interactions that fix the position of Cu1 coordinating histidines.

### 3.3. The Revision of the Role of His165 Located in the T3 Tunnel in the 2D Laccases Functioning

Most fungal 3D laccases oxidize 2.6-DMP, SGZ (syringaldazine), and ABTS at acidic conditions (pH 3.0–4.0). To date, only a few 3D laccases capable of oxidizing substrates of phenolic origin at neutral and alkaline pH have been discovered or engineered [21,22]. On the other hand, 2D laccases SLAC, Sila, Ssl1, SvSL, and SgfSL oxidize ABTS at acidic pH and 2.6-DMP at alkaline pH [18,23,24,25,26]. 

Earlier, we have shown that copper ions environments (excluding the 1st coordination sphere) in 2D and 3D laccases are different. It has been demonstrated that TNC copper ions of 3D laccases are surrounded by hydrophobic amino acid residues and only a few polar residues are located in these regions. In constrast, most of the residues close to TNC copper ions in 2D laccases are polar [26].We have proposed that due to the polar amino acid environment of TNC copper ions, 2D laccases are capable of oxidizing substrates at alkaline pH.

Comparison of the reaction rates of 2.6-DMP oxidation by mutants with replacements in the SBP, T3, and T2 tunnels, revealed an interesting pattern. Residue substitutions in T2 and T3 tunnels (Arg240His, His165Ala) did not affect the pH_opt_ of 2.6-DMP oxidation by SgfSL. However, the residual reaction rate of the His165Ala variant decreased slower with decreasing pH than that of SgfSLwt and Arg240His. The double mutant His165Ala/Arg240H retained an increased residual reaction rate at slightly acidic and neutral conditions. We propose that, due to mutation His165Ala, SgfSL loses the side group that might be protonated at alkaline pH. As a result, it loses the pH-selectivity, and its residual reaction rate at acidic and neutral conditions becomes higher. Our hypothesis is supported by the shift of pH_opt_ of 2.6-DMP oxidation by His165Ala/Met199Gly variant-pH 6.5 over pH 8.0 and pH 9.0 as for single mutant forms (Table 4). Apparently, in the absence of His 165 side group under conditions of an increased electron flux (due to replacing Met199Gly), shifting the pH_opt_ to the acidic region provides sufficient proton flux for oxidation 2.6-DMP by the His165Ala/Met199Gly variant.

The imidazole ring of His 165 in the SgfSL structure is localized similarly to the carboxyl group of Glu 498 (3D laccase *Bacillus subtillis*, PDB id 1GSK) which participates in the proton assisted reductive cleavage of the O–O bond [27]. Based on the data presented above we suggest that His 165 and some polar amino acid residues located near TNC of 2D laccases can participate in protonation events and influence the activity of these enzymes at alkaline pH. The residual reaction rate of mutant forms Met199Ala, Met199Gly, Tyr230Ala, and Met199Gly/Arg240His at slightly acidic and neutral conditions are approximately the same as that of the His165Ala variant, most likely due to pH_opt_ shifting by 0.5-1 point (Table 4).

It is known that the T3 tunnel leading to the Cu3β ion of 3D laccases is involved in the transfer of protons and oxygen to TNC [27]. At the same time, the role of the T2 tunnel leading to Cu2 ion of TNC in the transfer of protons and the removal of water as a by-product is also commonly accepted [28]. However, there is still no reliable data on the mechanism by which water molecules pass through the Cu2 ion into the T2 tunnel. Earlier, we determined the participation of Arg 240 located in the T2 tunnel at a distance of 10 Å from Cu2 ion in the oxygen protonation events of SgfSL [19]. Substitution of Arg 240 for His increased the k_cat_ of SgfSL activity toward 2.6-DMP and ABTS more than 3-fold. In contrast, the replacement of Arg 240 for Ala significantly reduced the SgfSL activity for both substrates. In this work, we have obtained a double mutant form (His165Ala/Arg240His) to investigate the effect of the combining these mutations. The structure of this variant was solved with high resolution (1.3 Å), and analysis showed a continuous chain of water molecules from the enzyme surface to the Cu2 ion, whereas in the SgfSLwt the water chain was interrupted by the side group of Arg 240. Moreover, the water molecule closest to His 240 forms hydrogen bonds with this residue and with Asp 108, which is impossible in the SgfSLwt structure due to the side-chain of Arg 240. Substitution of Arg 240 to His leads to the interaction of polar amino acids (Asp 108 and some others) in the T2 tunnel with water molecules directly, and not through an intermediary (Arg 240). All these data can help to explain why the Arg240His replacement increases the rate of the SgfSL enzymatic reaction in both alkaline and acidic conditions. Based on the high-resolution structure and biochemical data, we propose that the transfer of protons to the TNC clarifies the role of the water molecules chain located in the T2 tunnel of laccase structures, and the removal of water as a reaction product can occur through the T3 tunnel but not the T2.

### 3.4. The Combination of the Mutation in SBP and T2 Tunnels Increased the SgfSL Activity at Alkaline pH

We hypothesized that mutant forms with a combination of the substitutions in SBP (Met199Gly) and T3 tunnel (His165Ala) or T2 tunnel (Arg240His) would possess increased activity. Indeed, the activity of Met199Gly/Arg240His variant toward 2.6-DMP increased more than 3-fold compared to Met199Gly (Table 2). When Arg 240 was replaced by His in the single SgfSL mutant, the k_cat_ of 2.6-DMP oxidation increased by 3-fold. The k_cat_ of 2.6-DMP oxidation for the Met199Gly variant is one order of magnitude higher than for SgfSLwt, probably due to optimizing the substrate orientation in the SBP. Apparently, in case of double mutant Met199Gly/Arg240His, an increase in the electron flux from the substrate (Met199Gly) was supplemented by a relevant increase of proton transfer to TNC (Arg240His). Combining Met199Gly and His165Ala mutations did not significantly increase the SgfSL activity, but led to a drastic shift in the optimal pH of 2.6-DMP oxidation to an acidic pH area. These results indicate that His 165 regulates oxygen access and participates in the proton transport of 2D laccases. Thus, we suggest that to create a high-active laccase mutant, it is necessary to match the rates of electron and proton fluxes.

The access of inhibitors to TNC occurs through the tunnels. It was previously shown that the inhibition of laccase catalysis by azide and cyanide ions is competitive with oxygen [29]. The inhibitors and oxygen may most likely penetrate TNC via the same T3 tunnel. The presence of positively charged histidine at the entrance may be more attractive to negatively charged ions (N_3_^−^, CN^−^). Indeed, the SgfSL variant with the replacement of His165Ala improved both oxygen accesses to TNC and resistance to inhibitor [18]. In the present study, the His165Ala/Met199Gly mutant retained practically the same high resistance to azide ions as His165Ala, and the resistance of His165Ala/Arg240His variant was 3-fold higher than that of the wild type laccase (Table 3).

## 4. Materials and Methods

### 4.1. Plasmid Construction

The pQE-30-based plasmid pQE-993nS carrying the gene encoding SgfSLwt deprived of the N-terminal signal sequence was used as a template for the PCR with a pair of corresponding mutagenic primers (replaced nucleotides are in italic and underlined) [30].

Met199Ala_For:5′-CACACCATCGTGTTCAATGAC*GCG*ACAATCAACAACAGGCCCGCC-3′
Met199Ala_Rev:5′-GGCGGGCCTGTTGTTGATTGT*CGC*GTCATTGAACACGATGGTGTG-3′
Met199Gly_For:5′-ACCCACACCATTGTGTTCAACGAC*GGT*ACGATCAACAACAGGCCC-3′
Met199Gly_Rev:5′-GGGCCTGTTGTTGATCGT*ACC*GTCGTTGAACACAATGGTGTGGGT-3′
Tyr230Ala_For:5′-TCATGATCACGCACGGCGAG*GCC*TACCACACCTTCCACAT-3′
Tyr230Ala_Rev:5′-ATGTGGAAGGTGTGGTA*GGC*CTCGCCGTGCGTGATCATGA-3′

Amplification of target template was carried out by KOD Hot Start DNA Polymerase (Novagen, Darmstadt, Germany) according to the manufacturer’s instructions and QuikChange^TM^ method protocols [31]. The resulting plasmids, including the plasmid with SgfSLwt gene, were used as templates for PCR to further amplify the genes of interest, and later clone them into pET32-Xa/Lic vector. To that end, the following primer pair, specific to the 5′ and 3′ end regions of the genes (For and Rev), were used (restriction sites are in italic, and underlined).

For:5′-AGATCTG*GGTACC*ATCGAGGGTCGTGCAGGAGCAGCGCCCGCCGGGGGA-3′
Rev:5′-GGCTGC*AAGCTT*TCAGTGAGCGTGCTCCTGCGGGT-3′

The obtained PCR products were digested with HindIII and KpnI (Sibenzyme, Moscow, Russia) and inserted into the pET32-Xa/Lic vector, restricted with the same enzymes.

Double mutant form His165Ala/Arg240His was constructed using the QuikChange^TM^ method, using pQE30_SgfSL_His165Ala plasmid as a template for PCR and mutagenic primers for Arg240His insertion, as described [19]. Double mutant forms His165Ala/Met199Gly and Met199Gly/Arg240His were constructed according to QuikChange^TM^ protocol using plasmid pET32-Xa/Lic_SgfSL_Met199Gly as a template for PCR and primers for insertion, as described earlier [18]. 

### 4.2. Purification of SgfSLwt and Mutants 

The *E. coli* strain BL21(DE3)/Rosetta (Qiagen, Hilden, Germany) was transformed with pET32-Xa_Lic/SgfSLwt or the same plasmid carrying the gene of SgfSL with different mutations (Met199Ala, Met199Gly, Tyr230Ala, His165Ala/Met199Gly, Met199Gly/Arg240His). Cells were grown at 37 °C in TB (Terrific Broth media) with shaking at 160 rev/min until OD_600_ = 1. The production of SgfSLwt and mutants was induced by adding isopropyl β-D-1-thiogalactopyranoside (IPTG) to a final concentration of 0.25 mM. Along with IPTG, CuSO_4_ was added to a final concentration of 1 mM. After induction, cells were incubated for 18 h with low shaking (50 rev/min) at 25 °C. The gene of SgfSL with double mutation His165Ala/Arg240His was expressed in *E. coli* strain M15[pREP4] using pQE-30_SgfSL_His165Ala/Arg240His as a plasmid for competent cell transformation. Cell cultivation and induction of synthesis of the double mutant were performed as mentioned above, but using LB media. 

Cells were collected by centrifugation at 7000× *g* for 25 min, suspended in buffer A (20 mM phosphate buffer pH 7.4 containing 0.5 M NaCl and 20 mM imidazole) with 0.5 mM phenylmethylsulfonyl fluoride (PMSF) and 200 ng/ml DNaseI, and disrupted by EmulsiFlex-C3 high-pressure homogenizer (Avestin, Ottawa, Canada). Cell debris was removed by centrifugation (30 min at 10,000× *g*), and the supernatant was loaded onto a column packed with Ni Sepharose®HP (GE Healthcare, Uppsala, Sweden) equilibrated with buffer A. The column was washed with buffer A, and the protein was eluted with a step gradient of buffer A with imidazole at a final concentration of 150 mM. Protein-containing fractions were collected and dialyzed against buffer for proteolysis (50 mM Tris-HCl, pH 8.0, 100 mM NaCl). Proteolysis was carried out for 16 h at room temperature by adding 1U of factor Xa protease (Sigma-Aldrich, Taufkirchen, Germany) per 1 mg of proteins and CaCl_2_ to 1 mM. Following metal affinity chromatography on the same column was performed to make proteins without thioredoxin N-terminal tail. At the final stage, SgfSLwt and variants were concentrated to 10–30 mg/ml and dialyzed against buffer B (50 mM H_3_BO_3_-NaOH, pH 9.0, 100 mM NaCl). Double mutant His165Ala/Arg240His was purified with asimilar protocol, except stage of proteolysis (only one step of column chromatography). 

### 4.3. Activity Assays 

#### 4.3.1. Kinetic Parameters of SgfSLwt and Mutants

The laccase activities were assayed by measuring the amount of oxidized substrates ABTS (2,2-azino-bis-(3-ethylbenzthiazoline-6-sulfonate, Sigma), 2.6-DMP (2,6-dimethoxyphenol, Sigma), K_4_[Fe(CN)_6_] (Sigma) using Cary 100 UV-Visible Spectrophotometer.

The optimal pH of SgfSLwt and mutant variants activities were determined at 30 °C using the universal 50 mM Britton–Robinson (BR) buffer within the pH range 6.5–9.5 for 2.6-DMP, 2.5–4.5 for ABTS, and 3.0–4.5 for K_4_[Fe(CN)_6_]. The reaction mixture (1 mL) contained 1 mM of 2.6-DMP or 0.5 mM of ABTS or 1 mM of K_4_[Fe(CN)_6_] and the enzyme variants (0.25–12 μg). The laccase activity was determined as the amount of 2.6-DMP or ABTS or K_4_[Fe(CN)_6_] oxidized at 30 °C for 1 min. The kinetic parameters for all proteins were determined at the optimal pH using substrate range 0.1–3 mM, 0.0625–1.5 mM, 0.0625–1.5 mM for 2.6-DMP, ABTS, and K_4_[Fe(CN)_6_], respectively. All experiments were performed in triplicate.

The resulting curves fitted the Michaelis–Menten equation by nonlinear least-squares regression with SigmaPlot 11.0. In calculating the kinetic parameters, it was taken into account that the enzymes are homotrimers. Molar extinction coefficients used to determine the maximal velocity of substrate oxidation were ε_469_ = 49600 M^−1^ cm^−1^ for 2.6-DMP, ε_420_ = 36,000 M^−1^ cm^−1^ for ABTS and ε_420_ = 1020 M^−1^ cm^−1^ for K_4_[Fe(CN)_6_] [32,33,34].

#### 4.3.2. Inhibition by Sodium Azide

The inhibition effect of sodium azide was analyzed by measuring the residual activity of SgfSLwt and mutants after adding 10 mM of NaN_3_. The measurement was carried out at 30 °C in 50 mM BR buffer with optimal pH, constant amounts of enzymes, and 0.5 mM of ABTS. The residual activity was calculated, taking the initial activity of the enzymes without NaN_3_ as 100%. All experiments were performed in triplicate.

#### 4.3.3. Optimal Temperature for ABTS Oxidation, Thermal Stability, and Absorbance Change Determination after Heat Treatment of SgfSLwt and Mutants

The optimal temperature values of the laccases activity were determined in 50 mM BR buffer at a temperature ranging from 30 to 90 °C using 0.5 mM of ABTS as a substrate and 0.3–1 µg of enzymes (depends on enzyme activity). 

The thermal stability of laccase variants was investigated using ABTS as the substrate by pre-incubation of the enzyme solutions for 30 min at temperature 80 °C. After high-temperature incubation, proteins were immediately chilled on ice and centrifuged for 1 min at the maximum speed of a benchtop mini spin centrifuge. Residual activities were calculated by taking the initial activity of the enzyme before high temperature incubation as 100%. All experiments were performed in triplicate.

The UV visible spectra of laccases after incubation at 80 °C were determined using Cary 100 UV-Visible Spectrophotometer. The samples at concentration 3 mg/mL were heated for 5 min at 80 °C and centrifuged for 30 s at 11,700× *g* for cooling and condensate precipitation. The centrifuged samples were analyzed by spectrophotometer with λ range 190–700 nm using absorption mode. After data collection, samples were returned to the thermostat, and the procedure was repeated 3 times (up to 20 min). The residual intensity of Cu1 ion absorption peak after incubation at 80 °C was calculated, taking the intensity before heating as 100%.

#### 4.3.4. Decolorization of Dyes

Malachite green (MG) and indigo carmine (IC) were used as industrial important dyes for decolorization experiments [35]. The reaction mixtures for all proteins contained 1.42 µg of enzymes, 50 µM of MG or IC, 50 mM BR buffers with an optimal pH of dye decolorization, and 0.5 mM ABTS. The optimal pH of dyes decolorization by Met199Ala and SgfSLwt is pH 5.0 and by mutant Met199Gly and Tyr230Ala-6.5. All reactions were performed on a 1 ml scale at 30 °C in the light-free thermostat. Decolorization rates were detected by scanning from 190 nm to 700 nm using the UV-visible spectrophotometer. decolorization rates (D) were calculated by the formula: D = ((A_0_ − A_1_)/A_0_)) × 100%, where A_0_ and A_1_ are initial absorbance and absorbance after a certain period of time at maximum absorbance wavelengths depending on dye type. All experiments were performed in triplicate.

### 4.4. Crystallization and Crystallography

Crystallization experiments were performed at different temperatures (23 °C–37 °C) using the hanging-drop vapor-diffusion method on siliconized glass cover slides in Linbro plates (Molecular Dimensions, Sheffield, UK). Crystals of laccase variants for X-ray analysis were obtained using a micro-seeding strategy. Microcrystals of laccase mutants were grown using 20% *v/v* PEG Smear High, 0.1 M Bicine, pH 9.3 (condition #21 of BCS-1 from Molecular Dimensions, Sheffield, UK) as precipitant. The seed stock was prepared using the original article’s technique [36]. Prepared microcrystal solutions were used as seed stock crystals for further crystallization trials.

Crystallization drops were made by mixing 1.2 µL of protein at concentration 5–15 mg/mL, 0.3 µL of seed stock crystals and 0.9 µL of reservoir solution consisting of 15% *v/v* PEG Smear High, 0.15 M Ammonium acetate, 0.1 M Sodium citrate pH 5.0 (condition #31 of BCS-1 from Molecular Dimensions, Sheffield, UK). Except for His165Ala/Met199Gly mutant, a single crystal was flash cooled after soaking in a solution consisting of 20% PEG 4000, 20% glycerol, 0.1 M Na-acetate pH 4.5 as cryo-solution to collect the diffraction data. The diffraction data for His165Ala/Met199Gly crystals were collected at room temperature.

Diffraction data were collected on the beamline BL14.1 at the BESSY II electron storage ring (Berlin, Germany), ID30B at the ESRF electron storage ring (Grenoble, France), and home source Rigaku XtaLAB Synergy-S laboratory system (The Woodlands, Texas, USA). [37,38,39]. Synchrotron data were processed and merged using the XDS package [40]. Home source data were processed and merged using the CrysAlis PRO software [39]. Crystallographic data statistics are summarized in Table 1.

The structures were determined by molecular replacement with Phaser [41] using the structure of 2D laccase from *S. griseoflavus* determined at 1.8 Å resolution (PDB id 6S0O) as a search model. Water molecules and metal ions were removed from the model. The initial model was subjected to crystallographic refinement with REFMAC5 [42]. Manual rebuilding of the model was carried out in Coot [43]. The final cycle with an occupancy refinement of the copper ions was performed in Phenix [44]. Data and refinement statistics are summarized in Table 1. The atom coordinates and structure factors have been deposited in the Protein Data Bank. Figures were prepared using PyMOL [45].

## 5. Conclusions

Engineering of the substrate-binding pocket in combination with the tuning of the TNC improves the activity of SgfSL under neutral or alkaline conditions. The Met199Gly/Arg240His is a prospective 2D laccase variant with the highest activity under alkaline conditions. Besides, along with regulating oxygen access to TNC, the His 165 side chain may be required as donor/acceptor of protons in 2D laccases. The results show that protein engineering can overcome some of the limitations of low activity of bacterial laccases and allow the creation of biocatalysts with practical industrial applications. Optimizing a substrate-binding pocket with the additional tuning of the oxygen reduction process represents the next engineering step towards creating a laccase with the desired properties.

## Figures and Tables

**Table 1:** Crystallographic data collection and refinement statistics.

	Met199Ala	Met199Gly	Tyr230Ala	His165Ala/ Met199Gly	His165Ala/ Arg240His	Met199Gly/ Arg240His
**Data collection**						
Space group	P 2_1_	P 2_1_	P 2_1_	P 2_1_	P 2_1_	P 2_1_
Cell parameters:						
a,b,c (Å)	74.98, 94.12, 119.73	75.18, 94.36, 119.79	77.26, 94.98, 116.27	76.23, 94.59, 120.87	74.76, 94.29 120.48	74.06, 93.72, 119.23
α = γ = 90, β (°)	91.16	91.14	91.67	91.39	91.27	91.30
Collection temperature (K)	100	100	100	295	100	100
Resolution (Å)	50.00–1.75 (1.80–1.75) ^a^	50.00–1.75 (1.80–1.75) ^a^	50.00–1.60 (1.64–1.60) ^a^	25.00–2.20 (2.28–2.20) ^a^	50.00–1.30 (1.38-1.30) ^a^	25.0–1.85 (1.92–1.85) ^a^
Total No. Of reflections	1,135,633 (80,057)	1,148,856 (86,851)	1,241,259 (93,269)	317,814 (31,656)	2,743,665 (406,573)	484,277 (44,747)
No. Of unique reflections	167,091 (12,290)	168,209 (12,404)	220,573 (16,337)	85,543 (8557)	405,781 (64,031)	138,143 (13,817)
R_merge_ (%)	6.8 (110.1)	6.1 (172.2)	9.2 (103.5)	16.9 (59.7)	5.5 (135.8)	15.1 (63.6)
I/σ(I)	14.65 (1.53)	14.31 (1.02)	10.93 (1.55)	6.15 (1.36)	18.19 (1.28)	6.96 (1.06)
Completeness (%)	99.9 (99.6)	100.0 (100.0)	99.9 (99.9)	98.1 (99.3)	99.1 (97.0)	99.2 (98.0)
CC_1/2_	0.99 (0.64)	0.99 (0.50)	0.99 (68.3)	0.98 (0.52)	1.00 (0.55)	0.98 (0.51)
Redundancy	6.79 (6.51)	6.83 (7.00)	5.63 (5.71)	3.7 (3.7)	6.76 (6.35)	3.5 (3.2)
**Refinement**						
Resolution (Å)	47.24–1.75 (1.77–1.75)	47.18–1.75 (1.80–1.75)	47.10–1.60 (1.62–1.60)	24.74–2.20 (2.23–2.20)	47.41-1.30 (1.34-1.30)	24.68–1.85 (1.87–1.85)
No. reflections	167,072 (5530)	168,176 (12,756)	220,558 (7050)	85,458 (3541)	405,693 (12,826)	138,068 (4315)
R_work_ (%)	13.18 (21.64)	16.58 (31.81)	13.53 (30.44)	15.73 (23.87)	13.26 (31.20)	18.32 (31.76)
R_free_ (%)	16.93 (29.38)	20.13 (31.71)	16.53 (33.62)	18.85 (25.93)	15.69 (34.01)	22.35 (37.02)
R.m.s. deviations						
Bond lengths (Å)	0.006	0.007	0.006	0.007	0.006	0.008
Bond angles (°)	0.781	0.901	0.888	0.890	0.867	0.968
Ramachandran plot (%)						
Most favored	98.37	98.00	98.55	98.18	98.79	97.82
Additionally allowed	1.63	2.00	1.45	1.82	1.21	2.18
Generously allowed	0.00	0.00	0.00	0.00	0.00	0.00
PDB ID	7PFR	7PES	7PEN	7PU0	7PUH	7PTM

^a^ Values in parentheses are for the highest resolution shell.

**Table 2:** Kinetic constants and pH_opt_ of ABTS, 2.6-DMP, and K_4_[Fe(CN)_6_] oxidation by SgfSL-mutant forms and SgfSLwt. Kinetic constants for His165Ala and Arg240His variants are in Table S1. The values of kinetic constants of mutant forms are colored relative to the kinetic parameters of the wild-type protein (SgfSLwt):parameters indicating improved performance of the variants relative to the SgfSLwt are highlighted in orange, while those showing impaired performance than the SgfSLwt are marked in blue, as depicted in the colored scale shown below.

Substrate	Protein	pH_opt_	K_m_(mM)	k_cat_ (s^−1^)	k_cat_/K_m_ (s^−1^ mM^−1^)
ABTS	SgfSLwt	4.0	0.36 ± 0.07	15 ± 1	42
	Met199Ala	4.0	0.32 ± 0.05	33 ± 2	102
	Met199Gly	4.0	0.20 ± 0.06	45 ± 7	226
	Tyr230Ala	3.5	0.17 ± 0.04	29 ± 3	169
	His165Ala/Met199Gly	3.5	0.09 ± 0.01	9 ± 0	95
	Met199Gly/Arg240His	3.5	0.10 ± 0.015	20 ± 1	203
	His165Ala/Arg240His	3.5	0.075 ± 0.03	6 ± 0	74
2.6-DMP	SgfSLwt	9.0	0.32 ± 0.08	0.35 ± 0.02	1.09
	Met199Ala	8.0	0.83 ± 0.08	1.30 ± 0.05	1.55
	Met199Gly	8.0	0.67 ± 0.12	3.39 ± 0.17	5.07
	Tyr230Ala	8.5	0.76 ± 0.03	1.68 ± 0.07	2.22
	His165Ala/Met199Gly	6.5	0.28 ± 0.05	0.31 ± 0.01	1.11
	Met199Gly/Arg240His	8.5	0.25 ± 0.02	4.18 ± 0.09	16.51
	His165Ala/Arg240His	9.0	0.15 ± 0.016	0.11 ± 0.004	0.74
K_4_[Fe(CN)_6_]	SgfSLwt	4.0	0.10 ± 0.02	38 ± 2	383
	Met199Ala	4.0	0.35 ± 0.02	54 ± 1	155
	Met199Gly	4.0	0.25 ± 0.02	66 ± 2	261
	Tyr230Ala	4.0	0.31 ± 0.01	86 ± 1	278
	His165Ala/Met199Gly	4.0	0.24 ± 0.04	35 ± 2	144
	Met199Gly/Arg240His	4.0	0.17 ± 0.02	48 ± 3	279
	His165Ala/Arg240His	4.0	0.18 ± 0.02	29 ± 1	160

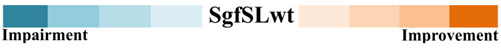

**Table 3:** The residual activity of SgfSLwt and mutants toward ABTS in the presence of 10 mM NaN_3_.

Protein	The Residual Activity (%) in the Presence of 10 mM of NaN_3_
SgfSLwt	23 ± 1
Met199Ala	13 ± 2
Met199Gly	20 ± 1
Tyr230Ala	20 ± 3
His165Ala/Met199Gly	116 ± 3
His165Ala/Arg240His	78 ± 5
Met199Gly/Arg240His	25 ± 1

**Table 4:** The relative rates of 2.6-DMP oxidation by the wild-type and mutant forms of SgfSL. The maximum rate in each raw (Vm, μmol sec^−1^ μg^−1^) was taken as 100%. The numerical values of Vm are shown in the last column.

Protein	Reaction Rate (%) at Different pH							Vm
	6.5	7.0	7.5	8.0	8.5	9.0	9.5	
SgfSLwt	3.95	11.94	27.62	50.47	88.68	100.00	65.73	1.1 × 10^−2^
Arg240His	3.62	7.77	11.63	36.05	75.58	100.00	82.56	3.3 × 10^−2^
His165Ala	9.44	25.42	46.25	73.37	96.97	100.00	76.39	6.0 × 10^−3^
His165Ala/Arg240His	12.50	25.00	45.83	75.00	87.50	100.00	95.83	2.8 × 10^−3^
Met199Ala	18.27	53.85	81.73	100.00	92.31	63.46	-	2.3 × 10^−2^
Met199Gly	36.36	79.68	97.33	100.00	68.98	37.97	-	8.3 × 10^−2^
Tyr230Ala	14.38	33.56	60.96	90.41	100.00	82.88	-	3.7 × 10^−2^
His165Ala/Met199Gly	100.00	64.71	58.82	51.47	41.18	39.22	-	6.9 × 10^−3^
Met199Gly/Arg240His	13.53	30.96	60.32	80.50	100.00	91.74	69.72	7.7 × 10^−2^

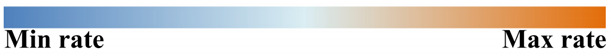

## Supplementary Materials

The following are available online at https://www.mdpi.com/article/10.3390/ijms23010065/s1.

## Abbreviations

TNC	TriNuclear Center
3D	Three-domain
2D	Two-domain
SgfSL	*Streptomyces griseoflavus* Small Laccase
