# Supplementary material for: Engineering the Catalytic Properties of Two-Domain Laccase from Streptomyces griseoflavus Ac-993
Source: Int J Mol Sci. 2021 Dec 22;23(1):65. doi: 10.3390/ijms23010065 (PMC8744557; doi:10.3390/ijms23010065)
Supplement: Supplementary file 1 [file ijms-23-00065-s001.zip › ijms-1474995-supplementary.pdf]

Table S1. Kinetic parameters of 2.6-DMP oxidation by His165Ala and Arg240His mutant forms.

substrate	protein	pH optima	Km (mM)	kcat (s^{-1})	kcat/Km (s^{-1} mM^{-1})
2.6-DMP	SgfSLwt	9.0	0.32 ±0.08	0.35 ±0.02	1.09
	His165Ala	9.0	0.18 ±0.03	0.19 ± 0.006	1.06
	Arg240His	9.0	1.09 ±0.15	1.28 ±0.06	1.17

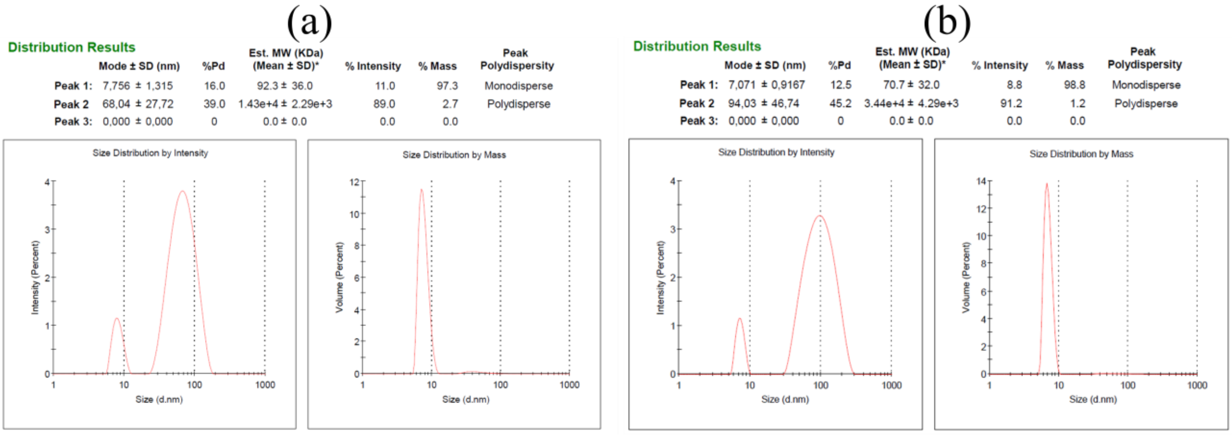

Figure S1. Dynamic light scattering peaks and reports of SgfSLwt (a) and Met199Gly (b) samples after the high-temperature incubation.
